# Effects of hypertension on the outcomes of COVID-19: a multicentre retrospective cohort study

**DOI:** 10.1080/07853890.2021.1931957

**Published:** 2021-06-03

**Authors:** Li Zhong, Yuting Wu, Jinghua Gao, Jinxia Zhang, Qifeng Xie, Huang He, Jingjing Ji, Zheying Liu, Conglin Wang, Zhifeng Liu

**Affiliations:** a Department of Critical Care Medicine, The First Affiliated Hospital, Guizhou University of Chinese Medicine, Guiyang, China; b Department of Respiratory Medicine, General Hospital of Southern Theater Command of PLA, Guangzhou, China; c Department of Critical Care Medicine, General Hospital of Southern Theater Command of PLA, Southern Medical University, Guangzhou, China; d Cardiovascular Department, General Hospital of Southern Theater Command of PLA, Guangzhou, China; e Department of Urology Surgery, General Hospital of Southern Theater Command of PLA, Guangzhou, China; f Department of Anesthesiology, General Hospital of Southern Theater Command of PLA, Guangzhou, China; g Key Laboratory of Hot Zone Trauma Care and Tissue Repair of PLA, General Hospital of Southern Theater Command of PLA, Guangzhou, China

**Keywords:** COVID-19, hypertension, clinical characteristics, mortality, age

## Abstract

**Objectives:** Hypertension is thought to be a contributor to mortality in coronavirus disease 2019 patients; however, limited clinical data on the outcomes of COVID-19 in patients with hypertension are available.

**Methods:** This study was designed to confirm whether hypertension affects the outcomes of COVID-19.

**Results:** A total of 983 patients with COVID-19 (female, 48%; male, 52%) were enrolled. Significantly higher odds of 60-day mortality (*p* = .017) were observed in the hypertensive group. In the hypertensive group, even after adjustment in multivariate analysis, the subgroup of patients 70 years old and older had higher 28-day mortality and total 60-day mortality rates than the other age subgroups (both*p* < .05). A total of 297 (89%) COVID-19 patients with hypertension survived, and 35 (11%) died. In addition, compared with hypertensive patients who survived COVID-19, non-survivors had more pre-existing conditions, including cardiovascular diseases and stroke, higher blood pressure on admission, more severe inflammation, and more liver and kidney damage.

**Conclusion:** Hypertension does not affect the outcome of COVID-19, which is different than the conclusions drawn in other studies. However, the 28-day mortality and total 60-day mortality rates of hypertensive patients (age ≥ 70) with COVID-19 were significantly elevated, and compared with the group of survivors, non-surviving COVID-19 patients with hypertension were older, had more basic diseases and had a more severe clinical condition.

## Introduction

In recent months, coronavirus disease 2019 (COVID-19) has posed a substantial threat to human health worldwide and has imposed a major burden on the global health care system [1,2]. Some studies suggested that basic diseases, of which hypertension was the most common [3,4], may increase the risk of mortality in COVID-19 patients. There are reports of associations between the death of COVID-19 patients and hypertension [5,6]; however, other studies have shown that hypertension is not related to the prognosis of COVID-19 and that there was insufficient evidence supporting a significant effect of hypertension on mortality due to COVID-19.

We designed a retrospective, multicentre cohort study involving four designated treatment centres in China, including in Wuhan. We obtained the hospital records of COVID-19 patients and extracted the prognostic indicators. In this study, we explored the influence of hypertension on the survival of COVID-19 patients to provide a reference for further clinical studies of hypertensive patients with COVID-19.

## Methods

### Study population

This was a retrospective, multicentre cohort study that included inpatients from four designated treatment centres in China, including Wuhan Taikang Tongji COVID-19 Specialized Hospital, Wuhan Huoshen Mountain COVID-19 Specialized Hospital, Department of Nephrology of General Hospital of Central Theatre Command of PLA (specialized department for the treatment of COVID-19 patients at that time), Department of Neurology of Xinhua Hospital of Hubei Province (specialised department for the treatment of COVID-19 patients at that time). They all are Class 3 A Hospital in Wuhan of China. All patients had confirmed COVID-19 according to the World Health Organization (WHO) interim guidance between January 2020 and March 2020 [7]. This study was approved by the Ethics Committee of the General Hospital of the Southern Theatre Command(Number: Hospital Ethics[2020]-8), and the need to obtain informed consent was waived.

The inclusion criteria were as follows: (1) adults ≥18 years old; (2) laboratory (RT-PCR) confirmation of severe acute respiratory syndrome coronavirus 2 (SARS-CoV-2) infection in throat swab, sputum and/or lower respiratory tract samples; and (3) in-hospital treatment ≥ 72 h. The exclusion criteria were as follows:(1) existing evidence of other aetiologies of pneumonia, including but not limited to influenza A virus infection, influenza B viral infection, bacterial pneumonia, fungal pneumonia, and non-infectious pneumonia; (2) pregnancy or breastfeeding; and (3) missing key clinical information.

### Procedures

Basic clinical information, underlying diseases, diagnosis, clinical classification of COVID-19, inflammatory and organ function parameters on admission, including the white blood cell (WBC) count, procalcitonin (PCT) level, C-reactive protein (CRP) level, interleukin-6 (IL-6) level, liver function parameters, renal function parameters, blood lactate acid concentration, oxygenation index, Acute Physiology and Chronic Health Evaluation II (APACHE II) score and SOFA score, were recorded in this study. We compared the clinical characteristics on admission; the primary clinical outcomes, including 28-day and 60-day in-hospital mortality rates, the duration of hospitalization; and the duration of the disease course between the hypertensive and non-hypertensive groups. The hypertensive group was further divided into the surviving group and non-surviving group. The clinical characteristics were further compared between these two groups to identify the factors associated with survival.

The severity of COVID-19 was classified according to the Chinese Recommendations for Diagnosis and Treatment of Novel Coronavirus (SARS-CoV-2) infection (Trial 7th version) published by the National Health Commission of China [8] into 4 groups: mild, moderate, severe, and critical.

### Statistical analysis

The categorical data are summarized as frequencies and percentages, and intergroup comparisons were performed using χ2 test or Fisher’s exact test. Continuous variables are expressed as the medians and interquartile ranges. Continuous data with a Gaussian distribution were compared with Student’s t-test or one-way ANOVA, and variables with a non-Gaussian distribution were compared with the Mann–Whitney U-test. The patient endpoint event was death within 60 days after onset. The survival curve was drawn using the Kaplan–Meier method. Statistical analysis was performed using the SPSS Windows version 11.0 statistical package (SPSS Inc, Chicago, IL), and *P* values (two-tailed) less than .05 were considered statistically significant.

## Results and discussion

### Demographics and characteristics

A total of 983 patients were enrolled in the final analysis (Supplementary figure 1). The median age was 61.0 years (IQR 49.0-70.0), 468 (48%) were female, and 515(52%) were male. COVID-19 patients had several comorbidities, including coronary heart disease, chronic kidney disease, diabetes, chronic obstructive pulmonary disease, stroke, carcinoma, and other (Table 1). Hypertension (34%) was the most common comorbidity, followed by diabetes (15%) and coronary heart disease (11%). Based on the severity of COVID-19, patients were divided into four groups: 26(3%) were in the mild group, 434 (45%) were in the moderate group, 376 (39%) were in the severe group, and 133 (14%) were in the critical group. The baseline clinical characteristics were as follows: body temperature of 36.7 °C (IQR 36.4–37.3), pulse of 85.0 beats/min (IQR 78.5–96.0), respiration rate of 20.0 beats/min (IQR 18.0–21.0), blood pressure of 129.0 (IQR 118.0–140.0)/78.0 (70.0–85.0) mmHg, APACHE II score of 5.0 (IQR 3.0–8.0) and SOFA score of 2.0 (IQR 1.0–3.0) (Table 1).

**Figure 1.**
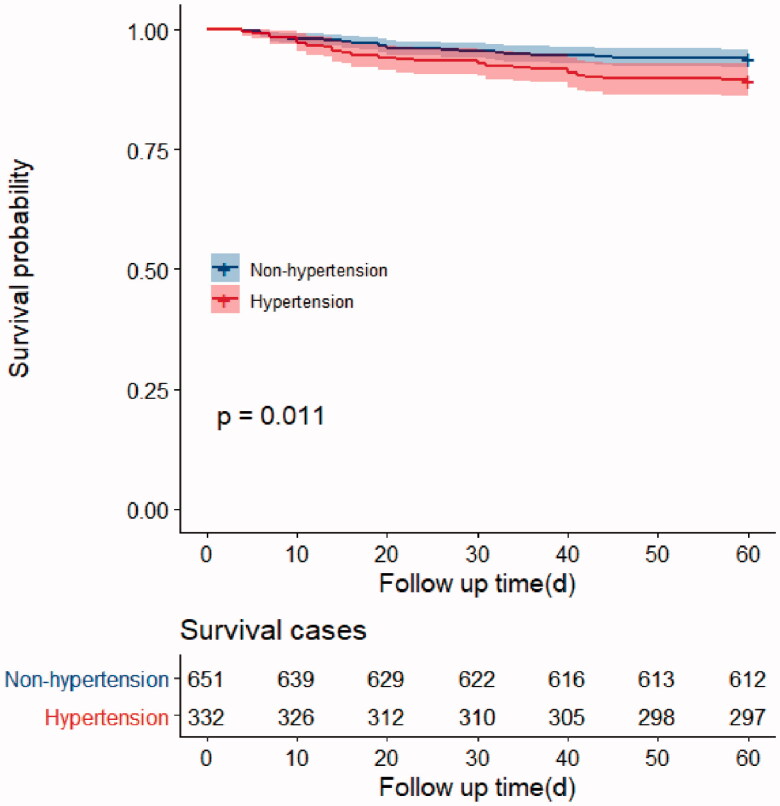
Survival curves of 60-day mortality rate in Hypertension group and Non-hypertension group

**Table 1.** Baseline characteristics of demographics, clinical and laboratory findings in Hypertension group and Non-hypertension group.

	Total (*N* = 983)	Hypertension group (*N* = 332)	Non-hypertension group (*N* = 651)	*p* Value
Demographics, clinical characteristics				
Age, years	61.0 (49.0–70.0)	72.0 (65.5–81.5)	58.0 (47.0–69.0)	<.001
Sex *N* (%)				
Male	515 (52%)	191 (58%)	324 (50%)	.021
Female	468 (48%)	141 (43%)	327 (50%)	
Coronary heart disease	105 (11%)	73 (22%)	32 (5%)	<.001
Chronic kidney disease	21 (2%)	16 (5%)	5 (1%)	<.001
Diabetes	148 (15%)	96 (29%)	52 (8%)	<.001
Chronic obstructive lung	21 (2%)	13 (4%)	8 (1%)	.006
Stroke	62 (6%)	45 (14%)	17 (3%)	<.001
Carcinoma	33 (3%)	15 (5%)	18 (3%)	.144
Otherdiseases	235 (24%)	108 (33%)	127 (20%)	<.001
Temperature (°C), median (IQR)	36.7 (36.4-37.3)	36.6 (36.4-37.0)	36.7 (36.4-37.3)	.009
Pulse (beats per min), median (IQR)	85.0 (78.5-96.0)	83.0 (78.0-90.0)	85.0 (78.0-96.0)	.046
Respiratory rate (breaths per min), median (IQR)	20.0 (18.0-21.0)	20.0 (18.5-22.0)	20.0 (18.0-21.0)	<.001
Systolic blood pressure, median (IQR)	129.0 (118.0-140.0)	127.0 (115.0-141.0)	128.5 (118.0-140.0)	<.001
Diastolic blood pressure, median (IQR)	78.0 (70.0-85.0)	76.0 (70.0-82.0)	78.0 (70.0-85.0)	<.001
Clinical Classifications *N* (%)				.142
Mild	26 (3%)	7 (2%)	19 (3%)	
Ordinary	434 (45%)	138 (42%)	296 (46%)	
Severe type	376 (39%)	126 (39%)	250 (39%)	
Critical type	133 (14%)	56 (17%)	77 (12%)	
APACHE II	5.0 (3.0-8.0)	7.0 (5.0-10.0)	5.0 (2.0-7.0)	<.001
SOFA	2.0 (1.0-3.0)	2.0 (1.0-3.0)	2.0 (0-3.0)	0.006
Laboratory findings, median (IQR)				
WBC, (1 × 109/L) 5.7 (4.4–7.4) 6.3 (4.8–8.0)			5.6 (4.3–7.4)	.034
NEU, (1 × 109/L)	3.7 (2.5–5.4)	4.5 (3.1–6.0)	3.6 (2.5–5.4)	.001
LYM, (1 × 109/L)	1.1 (0.7–1.6)	0.9 (0.5–1.4)	1.1 (0.7–1.6)	.004
MON, (1 × 109/L)	0.5 (0.3–0.6)	0.4 (0.3–0.6)	0.5 (0.3–0.6)	.828
PLT, (1 × 109/L)	190.0 (148.0–243.0)	176.0 (134.0–264.0)	191.0 (149.0–242.0)	.471
HGB, (g/L)	126.0 (114.0–138.0)	122.0 (106.8–135.3)	126.0 (114.0–138.0)	.018
FIB, (g/L)	4.1 (3.4–4.9)	4.3 (3.1–6.6)	4.1 (3.4–4.9)	.081
IL-6, (pg/ml)	11.3 (3.4–30.1)	24.7 (5.5–73.6)	10.7 (3.0–28.0)	.042
PCT, (ng/ml)	0.1 (0–0.1)	0.1 (0.1–0.2)	0.1 (0–0.1)	<.001
CRP, (mg/L)	13.9 (3.2–47.8)	44.6 (9.7–72.4)	12.7 (2.8–44.6)	<.001
ALT, (U/L)	23.0 (16.0–37.0)	27.0 (19.0–51.8)	23.0 (16.0–36.0)	.036
TBIL, (umol/L)	11.2 (8.3–14.7)	13.4 (10.7–18.7)	11.0 (8.1–14.4)	.099
DBIL, (umol/L)	3.1 (2.1–4.7)	4.3 (3.0–6.3)	3.0 (2.1–4.6)	.033
CREA, (µmol/L)	63.0 (50.4–79.0)	74.5 (58.3–89.2)	62.0 (50.0–76.3)	<.001

IQR, Inter-Quartile Range; WBC: White blood cell count; NEU: Neutrophil; LYM: Lymphocyte count; MON: Monocytes; PLT: Platelet count; HGB: Haemoglobin; FIB: Fibrinogen; IL-6: Interleutin-6; PCT: Procalcitonin; CRP: C-reactive protein; ALT: Alanine aminotransferase; TBIL: Total bilirubin; DBIL: Direct bilirubin; CREA: Creatine.

### Clinical characteristics of non-hypertensive versus hypertensive patients

The proportion of patients with hypertension in our population was 34%. When grouping our population according to the presence of hypertension, we observed that the group with hypertension was older and had more concomitant conditions, such as coronary heart disease, chronic kidney disease, chronic kidney disease, diabetes, chronic obstructive pulmonary disease, and stroke (Table 1). The prevalence of hypertension was higher in males than in females. Furthermore, a higher APACHE II score, higher SOFA score, more severe inflammation, and more organ damage were observed in the hypertensive group than in the non-hypertensive group (*p* < .05) (Table 1).

We found that there were significant differences in the odds of 60-day mortality and the total duration of the disease course in the hypertensive and non-hypertensive groups (*p* < .05) (Table 2). These differences might not have been due to hypertension. Accordingly, to verify whether the mortality rate in the hypertensive group was higher than that observed in the non-hypertensive group, we performed a multivariate analysis. As expected, there were no significant differences between the two groups in 28-day mortality, total 60-day mortality, hospitalization duration, or total disease course duration after adjusting for sex, age, APACHE II score, and SOFA score (*p* = .615, *p* = .791) (Table 2 and Supplementary table 2). Moreover, the results of the survival analysis showed a lower probability of survival at 60 days in the hypertensive group than in the non-hypertensive group (*p* = .011) (Figure 1).

**Table 2.** Outcomes of non-hypertension and hypertension.

All cohort	Non-Hypertension (*n* = 651)	Hypertension (*n* = 332)	*p*-value	OR*(95%CI)	*p*-value*
28-day fatality			.147	0.785 (0.305–2.017)	.615
Survivor	622 (96%)	310 (93%)			
Non-survivor	29 (5%)	22 (7%)			
60-day fatality			.011	1.123 (0.478–2.637)	.791
Survivor	612 (94%)	297 (90%)			
Non-survivor	39 (6%)	35(11%)			
In-hospital days	23.0 (15.0-35.0)	26.0 (16.0-40.0)	.171	0.990 (0.972–1.008)	.281
Total course of disease^a^	39.0 (27.0-53.0)	41.0 (29.0-54.0)	.011	0.993 (0.977–1.009)	.383

*Adjusted: Sex, Age, APACHE II,SOFA; ^a^Total course of disease：Time from illness onset to death or discharge, days.

The hypertensive group was further divided according to age into two subgroups: patients <70 years old and patients ≥70 years old. After adjustment for sex, age, APACHE II score and SOFA score, both the 28-day mortality rate and the total 60-day mortality rate were significant different between the two subgroups (*p* < .05); the mortality rate was significantly higher in the subgroup of elderly patients over 70 years old among the population of COVID-19 patients with hypertension (Supplementary tables 1,3). Among COVID-19 patients without hypertension, there were no significant differences in the 28-day and 60-day mortality rates between the subgroups of patients aged <70 years and ≥70 years after adjustment for sex, APACHE II score, and SOFA score, showing that age is not a risk factor for mortality in patients with COVID-19 who do not have hypertension (Supplementary tables 1,3).

**Table 3.** Baseline characteristics of survivors and non-survivors in patients with hypertension.

	Total (*N* = 332)	survivors (*N* = 297)	Non-survivors (*N* = 35)	*p* Value
Demographics, clinical characteristics				
Age, years	68.0 (60.0–75.8)	67.0 (59.0–73.0)	79.0 (70.0–85.0)	<.001
Sex *N* (%)				.162
Male	191 (58%)	167 (56%)	24 (69%)	
Female	141 (43%)	130 (44%)	11 (31%)	
Coronary heart disease	73 (22%)	60 (20%)	13 (37%)	.022
Chronic kidney disease	16 (5%)	14 (5%)	2 (6%)	.681
Diabetes	96 (29%)	83 (28%)	13 (37%)	.256
Chronic obstructive lung	13 (4%)	12 (4%)	1 (3%)	.782
Stroke	45 (14%)	35 (12%)	10 (29%)	.015
Carcinoma	15 (5%)	14 (5%)	1 (3%)	.612
Other	108 (33%)	94 (32%)	14 (41%)	.268
Temperature (°C),median (IQR)	36.6 (36.3–37.1)	36.6 (36.3–37.0)	36.6 (36.3–37.3)	.862
Pulse (beats per min), median (IQR)	87.0 (80.0–98.0)	86.0 (80.0–98.0)	90.0 (79.5–100.0)	.432
Respiratory rate (breaths per min), median (IQR)	20.0 (19.0–23.0)	20.0 (18.0–22.0)	23.0 (20.0–28.0)	<.001
Systolic blood pressure, median (IQR)	132.5 (120.0–147.0)	132.0 (120.0,145.0)	138.5 (129.3–160.8)	.048
Diastolic blood pressure, median (IQR)	80.0 (72.0–87.8)	80.0 (72.0–88.0)	75.5 (68.0–85.0)	.044
Laboratory findings, median (IQR)				
WBC, (1 × 109/L) 6.0 (4.5–8.5) 5.8 (4.4–7.7)			9.2 (5.4–12.8)	<.001
NEU, (1 × 109/L)	4.0 (2.7–6.8)	3.9 (2.7–5.8)	8.2 (4.2–11.8)	<.001
LYM, (1 × 109/L)	1.0 (0.6–1.4)	1.1 (0.8–1.5)	0.6 (0.4–0.8)	<.001
MON, (1 × 109/L)	0.5 (0.3–0.6)	0.5 (0.3–0.6)	0.4 (0.2–0.6)	.084
PLT, (1 × 109/L)	187.0 (144.5–242.5)	196.0 (147.0–247.0)	153.5 (74.0–189.8)	<.001
HGB, (g/L)	124.0 (109.0–134.5)	124.5 (110.0–134.0)	118.0 (94.0–138.0)	.448
FIB, (g/L)	4.4 (3.4–5.4)	4.4 (3.4–5.4)	4.3 (3.0–5.6)	.507
IL-6, (pg/ml)	15.0 (4.0–46.7)	10.5 (3.0–33.0)	95.0 (42.7–179.0)	<.001
PCT, (ng/ml)	0.1 (0–0.2)	0.1 (0.1–0.2)	0.2 (0.1–0.3)	<.001
CRP, (mg/L)	28.2 (6.2–68.1)	18.7 (3.9–50.3)	65.8 (38.8–150.0)	<.001
ALT, (U/L)	25.0 (17.3–43.8)	25.0 (17.0–44.2)	24.0 (17.5–43.8)	.827
TBIL, (umol/L)	11.4 (8.8–15.0)	11.0 (8.6–14.4)	14.5 (11.6–21.7)	.001
DBIL, (umol/L)	3.3 (2.3–5.3)	3.0 (2.2–4.6)	6.4 (4.6–10.3)	<.001
CREA, (µmol/L)	69.2 (52.4–91.0)	68.7 (50.9–88.2)	78.8 (60.4–125.9)	.015

IQR: Inter-Quartile Range; WBC: White blood cell count; NEU: Neutrophil; LYM: Lymphocyte count; MON: Monocytes; PLT: Platelet count; HGB: Haemoglobin; FIB: Fibrinogen; IL-6: Interleutin-6; PCT: Procalcitonin; CRP: C-reactive protein; ALT: Alanine aminotransferase; TBIL: Total bilirubin; DBIL: Direct bilirubin; CREA: Creatine.

### Clinical characteristics of survivors versus non-survivors

Ultimately, 297 (89%) COVID-19 patients with hypertension survived, and 35 (11%) died. Subgroup analysis of the COVID-19 patients with hypertension in our study revealed that compared with survivors, non-survivors were older and had more complications, such as coronary heart disease, stroke, and higher blood pressure on admission. In addition, the level of inflammation and degree of organ damage in non-survivors were more severe than those in survivors (*p* < .05) (Table 3).

### Discussion

This retrospective cohort study identified the contribution of hypertension to the mortality of patients infected with SARS-CoV-2. Several conclusions were confirmed by our findings. First, we found that there was no difference in the mortality rate of COVID-19 patients based on the presence of hypertension after correction for basic characteristics. However, the mortality rate of COVID-19 patients with hypertension who were ≥70 years old was significantly higher than that of those who were < 70 years old, and compared with the survivors, patients with hypertension who did not survive were older, had more basic diseases and a more severe clinical condition.

A previous study found that at least one comorbidity was present in nearly 20–51% of patients [9], with hypertension being the most common comorbidity (12.6–38%) [10–13]. Indeed, the proportion of patients with hypertension (34%) in our population was comparable to those reported in previous publications. Hypertensive patients often have other underlying diseases such as coronary heart disease, and the prevalence of such conditions increases with increasing age [14]. Similarly, COVID-19 patients with hypertension are older and have more underlying diseases than those without hypertension. Previously, hypertension has been reported to be associated with an increase in unfavourable outcomes of COVID-19, including acute respiratory distress syndrome (ARDS), more severe COVID-19, admission to the ICU, and even mortality [9,15].

Recently, Leiva Sisnieguez CE *et al* commented on the study by Ruan *et al* [6]. They pointed out that hypertension was a risk factor for mortality in patients with COVID-19, although they did not perform a multivariate analysis to adjust for potential confounders [16]; therefore, their conclusions may have some limitations. As expected, our data showed that the 28-day and 60-day mortality rates of the hypertensive group and the non-hypertensive group were not significantly different after correction for sex, age, APACHE II score, and SOFA score, indicating that hypertension is not a risk factor for mortality in COVID-19 patients. However, the 28-day mortality rate and total 60-day mortality rate of hypertensive COVID-19 patients who were ≥ 70 years old were significantly elevated. In addition, non-survivors with hypertension were older, had more underlying diseases and had a more serious clinical condition than survivors with hypertension.

Another conclusion of our study was that the 28-day mortality and total 60-day mortality rates of hypertensive COVID-19 patients ≥70 years old were significantly higher than those of hypertensive COVID-19 patients <70 years old after adjustment for sex, APACHE II score, and SOFA score. Some studies have shown that age is a key risk factor for COVID-19-related mortality [17], but in our study, age was a key factor affecting mortality only in the hypertensive population. Our study provides a more precise understanding of the effects of age and hypertension on the outcomes of COVID-19.

Moreover, the results of the survival analysis showed a shorter survival time and more severe inflammation and organ damage in the hypertensive group than in the non-hypertensive group. Subgroup analysis of the COVID-19 patients with hypertension in our study revealed that inflammation and organ damage were more severe in non-survivors than in survivors. It may be that suffering from a chronic disease leads to a weakened innate immune response, and a severe viral infection induces systemic inflammatory response syndrome, which increases the risk of plaque rupture and thrombosis [18], vascular plaques are often present in patients with cardiovascular and cerebrovascular diseases. Another study showed that the deterioration of COVID-19 patients was related to the sharp increase in pro-inflammatory cytokines [19]^,^ such as IL-2, IL-7 and IL-6, the levels of which were reported to be closely correlated with the clinical outcomes of COVID-19. The increases in cytokine concentrations can lead to a “cytokine storm”, which may be the driving factor leading to acute lung injury, ARDS and multiple organ failure (MOF) [20]. Additionally, these cytokines are also interrelated with the progression of hypertension [21]. Therefore, we suggest that COVID-19 patients with hypertension are more likely to experience a more severe systemic inflammatory response, which in turn will lead to multiple complications, such as ARDS, disseminated intravascular coagulation (DIC), and multiple organ dysfunction syndrome (MODS), eventually leading to death.

This study has certain limitations. First, it was a multicentre retrospective cohort study, not all patients had all laboratory tests, which may reduce the reliability of the statistical analysis. Second, patients in the four centres are all in one region, and the overall number of cases needs to be further expanded, and the observation period needs to be extended further to observe the long-term effects. Third, the drugs and effects of blood pressure control in patients are not detailed enough. Our current research is mainly to answer the impact of hypertension as an underlying diseases on the short-term outcomes of patients with COVID-19. In the future, more data are needed to analyse the effects of hypertension control and antihypertensive drugs, as well as the possible effect of COVID-19 on the long-term hypertension control.

### Conclusion

Overall, our study showed that hypertension does not affect the outcome of COVID-19, which is different than the conclusions drawn in other studies. However, the 28-day mortality and total 60-day mortality rates of hypertensive patients (age ≥ 70) with COVID-19 were significantly elevated, and compared with the group of survivors, non-surviving COVID-19 patients with hypertension were older, had more basic diseases and had a more severe clinical condition. Our study provides a more precise understanding of the effects of hypertension and age on the outcomes of COVID-19.

## Ethics approval and consent to participate

This study was approved by the Ethics Committee of the General Hospital of the Southern Theatre Command (Number: Hospital Ethics[2020]-8), and the need to obtain informed consent was waived.

## Supplementary Material

Supplemental Material
