# Supplementary material for: Effects of hypertension on the outcomes of COVID-19: a multicentre retrospective cohort study
Source: Ann Med. 2021 Jun 3;53(1):770–6. doi: 10.1080/07853890.2021.1931957 (PMC8183539; doi:10.1080/07853890.2021.1931957)
Supplement: Supplemental Material [file IANN_A_1931957_SM7648.docx]

| **Supplementary table 1 Outcomes of different age groups in patients with hypertension** | | | | | |
| --- | --- | --- | --- | --- | --- |
|  | **Age(＜70)**  **(N=192)** | **Age(≥70)**  **(N=140)** | **P value** | OR* (95%CI) | P-value* |
| 28-day fatality |  |  | <0.001 | 0.123(0.017-0.895) | 0.039 |
| Survivor | 189(98%) | 121(86%) |  |  |  |
| Non-survivor | 3(2%) | 19(14%) |  |  |  |
| 60-day fatality |  |  | <0.001 | 0.233(0.064-0.844) | 0.027 |
| Survivor | 184(96%) | 113(81%) |  |  |  |
| Non-survivor | 8(4%) | 27(19%) |  |  |  |
| In-hospital days | 25.0(16.0-36.0) | 23.0(15.0-37.0) | 0.230 | 1.035(1.002-1.070) | 0.037 |
| Total course of disease ^a^ | 44.0(29.0-56.5) | 43.0(29.0-52.0) | 0.106 | 1.035(1.005-1.066) | 0.022 |
| **Outcomes of different age groups in patients with Non- hypertension** | | | | | |
|  | **Age(＜70)**  **（N=533）** | **Age(≥70)**  **(N=118)** | **P value** | OR* (95%CI) | P-value* |
| 28-day fatality |  |  | 0.001 | 0.925(0.220-3.888) | 0.915 |
| Survivor | 516(97%) | 106(90%) |  |  |  |
| Non-survivor | 17(3%) | 12(10%) |  |  |  |
| 60-day fatality |  |  | <0.001 | 1.605(0.427-6.027) | 0.484 |
| Survivor | 512(96%) | 100(85%) |  |  |  |
| Non-survivor | 21(4%) | 18(15%) |  |  |  |
| In-hospital days | 23.0(15.0-33.0) | 26.0(16.0-36.0) | 0.119 | 0.993(0.964-1.023) | 0.646 |
| Total course of disease ^a^ | 37.0(25.0-51.0) | 45.0(31.0-53.0) | 0.010 | 0.964(0.939-0.989) | 0.005 |
| *Adjusted: Sex , APACHE II,SOFA; ^a^ Total course of disease：Time from illness onset to death or discharge, days | | | | | |

| **Supplementary table 2 Outcomes of Non-hypertension and Hypertension**  **(*Adjusted: Sex, Age, APACHE II,SOFA)** | | | | |
| --- | --- | --- | --- | --- |
|  | OR* | 95%CI | | P*-value |
| 28-day fatality | 0.785 | 0.305 | 2.017 | 0.615 |
| Age | 0.952 | 0.934 | 0.969 | <0.001 |
| Sex | 0.389 | 0.241 | 0.629 | <0.001 |
| APCHEII | 0.894 | 0.826 | 0.967 | 0.005 |
| SOFA | 1.176 | 1.038 | 1.332 | 0.011 |
|  | | | |  |
| 60-day fatality | 1.123 | 0.478 | 2.637 | 0.791 |
| Age | 0.952 | 0.935 | 0.969 | <0.001 |
| Sex | 0.386 | 0.239 | 0.623 | <0.001 |
| APCHEII | 0.898 | 0.830 | 0.971 | 0.007 |
| SOFA | 1.190 | 1.046 | 1.353 | 0.008 |
|  | | | |  |
| In-hospital days | 0.990 | 0.972 | 1.008 | 0.281 |
| Age | 0.952 | 0.934 | 0.969 | <0.001 |
| Sex | 0.397 | 0.245 | 0.643 | <0.001 |
| APCHEII | 0.899 | 0.831 | 0.972 | 0.007 |
| SOFA | 1.177 | 1.041 | 1.331 | 0.009 |
|  | | | |  |
| Total course of disease | 0.993 | 0.977 | 1.009 | 0.383 |
| Age | 0.953 | 0.935 | 0.970 | <0.001 |
| Sex | 0.397 | 0.245 | 0.644 | <0.001 |
| APCHEII | 0.898 | 0.831 | 0.971 | 0.007 |
| SOFA | 1.178 | 1.042 | 1.333 | 0.009 |

| **Supplementary table 3 Outcomes of different age groups in patients with hypertension(*Adjusted: Sex , APACHE II,SOFA)** | | | | |
| --- | --- | --- | --- | --- |
|  | OR* | 95%CI | | P*-value |
| 28-day fatality | 0.123 | 0.017 | 0.895 | 0.039 |
| Sex | 0.939 | 0.392 | 2.252 | 0.889 |
| APCHEII | 0.769 | 0.669 | 0.884 | <0.001 |
| SOFA | 1.354 | 1.050 | 1.745 | 0.019 |
|  | | | |  |
| 60-day fatality | 0.233 | 0.064 | 0.844 | 0.027 |
| Sex | 1.010 | 0.423 | 2.412 | 0.981 |
| APCHEII | 0.769 | 0.668 | 0.885 | <0.001 |
| SOFA | 1.329 | 1.047 | 1.687 | 0.019 |
|  | | | |  |
| In-hospital days | 1.035 | 1.002 | 1.070 | 0.037 |
| Sex | 0.973 | 0.412 | 2.295 | 0.950 |
| APCHEII | 0.739 | 0.639 | 0.856 | <0.001 |
| SOFA | 1.271 | 0.995 | 1.625 | 0.055 |
|  | | | |  |
| Total course of disease | 1.035 | 1.005 | 1.066 | 0.022 |
| Sex | 1.022 | 0.433 | 2.409 | 0.961 |
| APCHEII | 0.730 | 0.628 | 0.849 | <0.001 |
| SOFA | 1.256 | 0.984 | 1.605 | 0.068 |
|  |  |  |  |  |
| **Outcomes of different age groups in patients with Non- hypertension(*Adjusted: Sex , APACHE II,SOFA)** | | | | |
|  | OR* | 95%CI | | P*-value |
| 28-day fatality | 0.925 | 0.220 | 3.888 | 0.915 |
| Sex | 0.835 | 0.420 | 1.661 | 0.607 |
| APCHEII | 0.740 | 0.658 | 0.832 | <0.001 |
| SOFA | 1.342 | 1.128 | 1.597 | 0.001 |
|  | | | |  |
| 60-day fatality | 1.605 | 0.427 | 6.027 | 0.484 |
| Sex | 0.828 | 0.416 | 1.647 | 0.590 |
| APCHEII | 0.744 | 0.661 | 0.837 | <0.001 |
| SOFA | 1.371 | 1.142 | 1.646 | 0.001 |
|  | | | |  |
| In-hospital days | 0.993 | 0.964 | 1.023 | 0.646 |
| Sex | 0.849 | 0.425 | 1.695 | 0.642 |
| APCHEII | 0.741 | 0.660 | 0.833 | <0.001 |
| SOFA | 1.344 | 1.130 | 1.597 | 0.001 |
|  | | | |  |
| Total course of disease | 0.964 | 0.939 | 0.989 | 0.005 |
| Sex | 1.024 | 0.502 | 2.091 | 0.958 |
| APCHEII | 0.751 | 0.668 | 0.844 | <0.001 |
| SOFA | 1.317 | 1.105 | 1.568 | 0.002 |

983 patients included

Hypertension group

N=332

Non- hypertension group

N=651

Survivors

(N=297)

Non- survivors

(N=35)

**Supplementary figure1: Flowchart of all excluded and included patients**
